# Fouling Mechanisms in the Clarification of 1,3-Propanediol Fermentation Broths by Membrane Processes

**DOI:** 10.3390/membranes15090276

**Published:** 2025-09-12

**Authors:** Hong Chen, Fu Yang, Qianyu Wang, Tianyu Zheng, Rongqing Zhou, Chongde Wu, Yao Jin

**Affiliations:** 1 College of Biomass Science and Engineering, Sichuan University, Chengdu 610065, China; 2022223085112@stu.scu.edu.cn (H.C.); 2022323080019@stu.scu.edu.cn (F.Y.); 2023223080024@stu.scu.edu.cn (Q.W.); 2023223080028@stu.scu.edu.cn (T.Z.); zhourqing@scu.edu.cn (R.Z.); cdwu@scu.edu.cn (C.W.); 2 Key Laboratory for Leather and Engineering of the Education Ministry, Sichuan University, Chengdu 610065, China; 3 National Engineering Research Center of Solid-State Manufacturing, Luzhou 646000, China

**Keywords:** 1,3-propanediol, fermentation broth, clarification, fouling mechanism, polymeric membranes

## Abstract

Membrane separation is an effective means of separating 1,3-propanediol (1,3-PD) from fermentation broth. However, systematic studies on membrane fouling behavior during this process are still limited. Therefore, this study systematically analyzed the membrane fouling behavior during the clarification of 1,3-PD fermentation broth using ultrafiltration/microfiltration and explored the effects of different membrane materials, pore sizes, and shear rates on permeation efficiency, target product recovery rate, and impurity removal rate. The results showed that the filtration of 1,3-PD fermentation broth was mainly dominated by cake formation, and the main foulant was identified as proteinaceous substances. Otherwise, increasing the shear rate adjacent to the membrane did not alter the membrane pore fouling mechanism, but it can disrupt the reversible fouling layer and reduce the growth rate of the fouling layer. Meanwhile, the results also indicated that the PES 100 kDa membrane exhibited the best overall performance with high recovery rate of 1,3-PD and excellent removal effects on impurities, significantly reducing the subsequent purification burden. This study provides more theoretical basis and data support for the optimization of membrane separation processes in 1,3-PD fermentation broth clarification.

## 1. Introduction

1,3-propanediol (1,3-PD) serves as a key monomer for the synthesis of bio-based polyester materials [1,2,3,4]. Its industrial production is confronted with the common technological challenge of separating complex fermentation broth systems [5]. The fermentation broth contains not only the target 1,3-PD but also various interfering components such as cell debris (0.5–5 μm), extracellular polysaccharides (10–300 kDa), residual proteins (20–150 kDa), and pigment substances [4,5,6,7,8]. These impurities not only increase the energy consumption of subsequent distillation processes but also lead to coking and blockage of distillation columns and catalyst deactivation [9]. Traditional pretreatment methods, such as centrifugation and flocculation [6,10], often suffer from incomplete solid–liquid separation and denaturation of heat-sensitive substances, which affect the efficiency of subsequent extraction. Therefore, the development of a pretreatment technology that combines high-efficiency separation with product protection has become a crucial breakthrough for enhancing the economic viability of 1,3-PD production.

Membrane separation technology has garnered industry attention due to its unique advantages in processing fermented products [11,12,13,14]. Microfiltration (MF) processes, which construct micrometer-scale pore barriers (0.1–10 μm), can non-destructively retain cell debris and suspended particles. Compared with traditional centrifugation, microfiltration offers higher operational continuity and lower solid-phase water content [11,15,16]. Ultrafiltration (UF)/nanofiltration (NF) [17] technologies, relying on nanoscale membrane pores (1–100 kDa), selectively separate dissolved macromolecules based on molecular size differences, achieving a separation precision 10–100 times higher than traditional filtration processes [12,18,19,20,21]. In fact, numerous studies have reported the effectiveness of membrane separation technology in separating 1,3-PD from fermentation broths, including ceramic fine UF membrane (cut-off 450 Da) [20], ceramic UF membrane (cut-off 8 kDa) [22], ceramic MF membranes (cut-off 0.14 μm), polypropylene MF membranes (cut-off 0.2 μm) [16], and polymeric NF membrane (cut-off 200–300 Da) [23]. Table 1 provides a detailed account of the application of membrane separation technology in 1,3-PD fermentation broth.

However, our understanding of membrane separation for 1,3-PD fermentation broth remains limited. Firstly, the interaction mechanisms between the complex components of the fermentation broth and membrane materials are not well understood, leading to a lack of theoretical guidance for predicting and controlling membrane fouling. Secondly, current research often employs empirical strategies, lacking systematic analysis based on mass transfer kinetics, as shown in Table 1. These issues restrict the large-scale application of membrane separation technology in 1,3-PD clarification.

Therefore, this study systematically investigates the applicability of membrane technology in the pretreatment of 1,3-PD fermentation broth, addressing the aforementioned engineering needs and technical challenges. By analyzing the effects of key parameters such as membrane material, pore size, and shear rates adjacent to the membrane on separation efficiency, and combining the kinetic analysis of membrane fouling layer formation, an optimized process scheme that balances technical feasibility and economic rationality is proposed. Findings of this work can provide more evidence for the clean production of 1,3-PD via biomanufacturing and offer practical references for the selection of membrane modules in industrial applications of clarification of 1,3-PD fermentation broth.

## 2. Materials and Methods

### 2.1. Source of Fermentation Broth

The fermentation broth used in the experiments of this thesis was obtained from the fermentation production of 1,3-PD using glycerol as a substrate by strain *K. pneumoniae S-01* in collaboration between. Jinkai Biotechnology Co., Ltd. in Chengdu, Sichuan, China and our laboratory. The fermentation broth was subjected to high-pressure steam sterilization, it was then fed directly into the membrane process without any clarification or centrifugation. Its initial 1,3-PD concentration was 82.80 g/L and the pH was 5.82.

### 2.2. Membrane Separation Setup and Process Parameters

**Figure 1:**
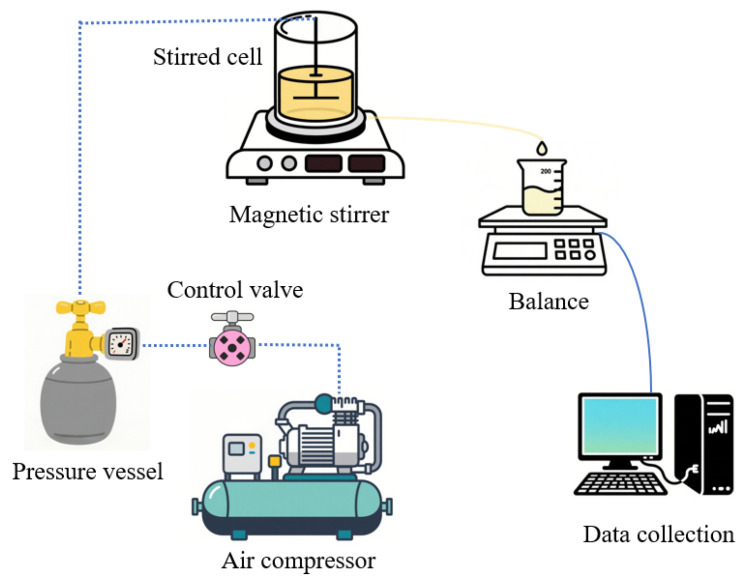
Diagram of dead-end filtration device.

The dead-end filtration device (Amicon UFSC40001, Millipore Corporation, Billerica, MA, USA) was self-assembled in our laboratory, shown in Figure 1. Such a setup corresponded to the setup previously employed by our research group [27,28,29]. A magnetically driven stirrer with adjustable speed at the bottom of the filter cell to generate a controllable shear force at the membrane surface: stirring speed was adjusted at 300, 600, and 900 rpm, corresponding to 57, 113, and 170 s^−1^. Permeate was collected in a vessel placed on a high-precision digital balance (accuracy 0.01 g) interfaced with a computer. An automated data-acquisition system recorded the cumulative permeate mass every 5 s, and the data were subsequently exported for analysis.

Four different hydrophilic membranes, polyethersulfone (PES), polypropylene (PP) with different pore sizes/MWCO (0.22 μm, 100 kDa or 50 kDa), were used in this work, and membrane characteristics details are provided in Table S1. They are denoted as PES-0.22, PP-0.22, PES-100k, and PES-50k. The membranes were treated before use (soaked in ultrapure water for 24 h, with water changed 3–5 times during this period) to remove impurities and additives from the manufacturing process.

### 2.3. Evaluation of Membrane Filtration Performance

#### 2.3.1. Permeate Flux

Permeate flux is a key indicator to measure membrane filtration performance, defined as the volume of fluid passing through a unit membrane area per unit time, and its calculation formula is

(1) $$J=\frac{V}{At},$$

where J represents permeate flux, usually in LMH (L/(m^2^·h)); V represents the volume of fluid passing through the membrane within time t, in liters (L); A is the effective filtration area of the membrane, in square meters (m^2^). In this experiment, the effective filtration area of the membrane module is 39.59 cm^2^; t is the filtration time, in hours (h).

#### 2.3.2. Filtration Efficiency

The Volume Reduction Rate (VRR) was used to characterize the separation effect of the membrane [30,31], and the calculation formula is as follows:

(2) $$VRR\;=\;\left(1\;-\;\frac{C_{p}}{C_{b}}\right)\;\times \;100\%$$

where $C_{p}$ is the concentration of the target solute or impurity in the permeate; $C_{b}$ is the initial concentration of the solute or impurity in the feed liquid. A higher VRR value indicates a stronger ability of the membrane to retain impurities and a better filtration effect. It is usually necessary to evaluate the membrane performance in combination with permeate flux to achieve an efficient membrane separation process.

### 2.4. Models for Analysis of Complex Process Behavior

Three models, the Resistance-in-series Model, Fouling Propensity Model, and Hermans and Bredee Model, were employed to deliver a comprehensive analysis of fouling behavior, based on the robust analytical framework previously established by our team [27,28,29,32,33,34].

#### 2.4.1. Resistance-in-Series Model

This model, as a classic analysis tool based on Darcy’s law, is mainly used to evaluate the magnitude and distribution characteristics of various fouling resistances during the filtration process. According to this model, the permeate flux in the filtration process can be calculated by Formula (3):

(3) $$J\;=\;\frac{∆P}{\mu R_{t}},$$

where J is the permeate flux (m^3^·m^−2^·s^−1^); ∆P is the transmembrane pressure difference (Pa); μ is the filtrate viscosity (Pa·s). In addition, $R_{t}$ is the total resistance during the filtration process (m^−1^), which can be calculated by Formula (4):

(4) $$R_{t}\;=\;R_{m}\;+\;R_{f}\;=\;R_{m}\;+\;R_{rf}\;+\;R_{irrf}\;=\;R_{m}\;+\;R_{rf}\;+\;R_{a}\;+\;R_{p},$$

where $R_{m}$ is the intrinsic resistance of the new membrane (m^−1^), calculated as in Formula (5); $R_{f}$ is the fouling layer resistance formed during microfiltration (m^−1^), which is composed of reversible fouling resistance ($R_{rf}$) and irreversible fouling resistance ($R_{irrf}$) (see Formulas (6) and (7) for details). Among them, $R_{rf}$ originates from concentration polarization ($R_{cp}$) and cake fouling ($R_{c}$) on the membrane surface, and such resistance can be removed by simple cleaning. R_irrf_, however, comes from adsorption ($R_{a}$) on the membrane surface or in pores and membrane pore blocking ($R_{p}$), which is difficult to eliminate by simple cleaning. The specific calculation formulas for each resistance are as follows:

(5) $$R_{m}\;=\;\frac{∆P}{\mu_{0}J_{0}},$$

(6) $$R_{f}=R_{t}-R_{m}=\frac{∆P}{\mu J}-\frac{∆P}{\mu_{0}J_{0}},$$

(7) $$R_{rf}=R_{f}-{\;R}_{irrf}=\frac{∆P}{\mu J}-\frac{∆P}{\mu_{0}J_{1}},$$

where $J_{0}$ is the pure water flux of the new membrane (m^3^·m^−2^·s^−1^), $J_{1}$ is the pure water flux of the fouled membrane after pure water cleaning (m^3^·m^−2^·s^−1^), and $\mu_{0}$ is the viscosity of water (Pa·s).

#### 2.4.2. Fouling Propensity Model

To quantitatively describe the growth law of fouling layer during filtration of 1,3-PD fermentation broth under different conditions, an exponential fouling model can be introduced. In this system, the evolution of total resistance with cumulative filtrate volume can be written as Formula (8) [35]:

(8) $$R_{t}\;={\;R}_{m}\exp(K_{0}V\;/\;A)$$

where V is the volume of filtered fermentation broth (m^3^), A is the effective membrane area (m^2^), and $K_{0}$ is the exponential fouling coefficient (m^−1^), an empirical constant representing the growth rate of fouling resistance. A larger value indicates faster deposition of impurities such as bacterial fragments, proteins, and polysaccharides in the fermentation broth on the membrane surface, leading to more severe fouling. The general filtration equation [36] is as follows:

(9) $$J\;=\;\frac{1}{A}\;\frac{dV}{dt}\;=\;\frac{∆P}{\mu R_{t}},$$

where t represents filtration time (s).

Substituting $R_{t}$ from Formula (9) into Formula (8), we obtain Formula (10):

(10) $$\frac{1}{A}\;\frac{dV}{dt}\;=\;\frac{∆P}{\mu R_{m}\exp(K_{0}V/A)},$$

Rearranging Formula (10) to obtain Formula (11), and integrating at t = 0, V = 0 and t = t, V = V, we get Formula (12):

(11) $$\int_{0}^{t}dt\;=\;\frac{\mu R_{m}}{∆PA}\;\int_{0}^{V}\exp\left(\frac{K_{0}V}{A}\right)dV$$

(12) $$V=\frac{A}{K_{0}}\ln(\frac{K_{0}∆P}{\mu R_{m}}\;t+1)$$

This model has been effectively verified in the analysis of membrane fouling mechanisms [37]. The value of $K_{0}$ is affected by various factors, including the composition of the fermentation broth (such as bacterial concentration, protein content, residual medium components), membrane separation operating conditions (such as transmembrane pressure, flow rate), membrane material properties, and membrane module structure.

#### 2.4.3. Hermans and Bredee Model

The membrane pore blocking model was proposed by Hermans and Bredee [38] in 1936. This model identifies the pore blocking mechanism [39,40] during the filtration process by plotting a double logarithmic graph of $d^{2}t/dv^{2}$ vs. dt/dv based on the flux decay behavior, thereby clarifying the interaction between particles in the 1,3-PD fermentation broth and the membrane. The slope of the linear regression fitting through this graph can determine the blocking mode, and its core formula is as follows:

(13) $$\frac{d^{2}t}{dv^{2}}=K\;{\left(\frac{dt}{dv}\right)}^{n}$$

where K is a constant, and n is the characteristic constant of the model. Since the experimental data of the cumulative permeate volume per unit membrane area v and time t are susceptible to noise interference, the n value calculated directly using Formula (13) may have deviations. Therefore, 4 derivative forms are usually used for linear fitting analysis.

In general, the value of n corresponds to different pore blocking mechanisms: n = 2.0 represents complete blocking, n = 1.5 for standard blocking, n = 1.0 indicates intermediate blocking, and n = 0 corresponds to cake formation. After integrating Formula (13), the following four linear equations can be obtained:

For complete blocking, n = 2:

(14) $$J\;=J_{0}\exp\left(-K_{b}t\right)$$

For standard blocking, n = 1.5:

(15) $$J\;=\frac{J_{0}}{{\left(\frac{K_{s}J_{0}}{2}t+1\right)}^{2}},$$

For intermediate blocking, n = 1.0:

(16) $$J\;=J_{0}\exp\left(-K_{i}t\right)$$

For cake filtration, n = 0:

(17) $$\frac{t}{v}\;=\;\frac{K_{c}}{2}\;v\;+\;\frac{1}{J_{0}},$$

where J is the permeate flux (unit: L/(m^2^·h)), $J_{0}$ is the initial permeate flux (unit: L/(m^2^·h)), t is the filtration time (unit: min), and $K_{b}$, $K_{s}$, $K_{i}$, $K_{c}$ are all model constants. By fitting the flux experimental data of 1,3-PD fermentation broth membrane filtration with Formulas (14)–(17) and comparing their regression coefficients, the most likely fouling mechanism can be inferred.

### 2.5. Rheological Measurements

The flow behavior of 1,3-PD fermentation broth (containing bacteria, proteins, polysaccharides, and residual glycerol) was determined using a modular precision rheometer at 25 °C. Considering the relatively low overall viscosity of the fermentation broth, a CP50-1 cone-plate system (diameter 50 mm, cone angle 1°) was selected for the test. The shear rates were set as 0.1, 0.3, 1, 3, 10, 30, 100, and 300 s^−1^ in sequence; when the shear rate reached 300 s^−1^, the filtrate viscosity value was recorded simultaneously to evaluate the viscosity stability in the high shear region.

If the fermentation broth does not show yield stress and its flow curve exhibits Newtonian or shear-thinning characteristics, it can be described by the power-law model:

(18) $$\tau \;=\;L\;{\dot{\gamma }}^{m}$$

where τ is the shear stress (Pa), L is the consistency coefficient (Pa·s^m^), $\dot{\gamma }$ is the shear rate (1/s), and m is the shear-thinning index.

When the fermentation broth shows obvious yield stress and gel-like consistency due to cell flocs or polysaccharide networks, the Herschel–Bulkley model is used for fitting [41,42]:

(19) $$\tau \;=\;\tau_{0}\;+\;L{\dot{\;\gamma }}^{m}$$

where $\tau_{0}$ is the yield stress (Pa).

### 2.6. Determination of Basic Properties

The pH value was directly measured using a pH meter (DZS-708-A, Leici, Shanghai, China). Zeta potential and particle size distribution were measured with a Malvern Mastersizer (ZEN3600 + MTP2, Malvern, UK). The protein content in the fermentation broth was quantitatively determined by the Coomassie Brilliant Blue G-250 dye binding method [43]. The cell and chromaticity contents of the fermentation broth were determined using an ultraviolet–visible spectrophotometer (TU-1901, Beijing, China).

### 2.7. Determination of 1,3-PD and Glycerol Contents

In this experiment, High Performance Liquid Chromatography (HPLC, Agilent Technologies, Waldbronn, Germany) was used for quantitative analysis of 1,3-PD and glycerol contents in the fermentation broth [44]. The specific chromatographic operating conditions are as follows: an organic acid ion exchange column was selected as the stationary phase for the separation process, and the column temperature was constantly controlled at 55 °C to ensure the stability of the separation effect; the mobile phase was a 5 mM sulfuric acid solution with a flow rate of 0.6 mL/min; the detection system was equipped with a differential detector, the single detection cycle was set to 20 min, and the injection volume for each time was precisely controlled to 10 μL.

### 2.8. Determination of Membrane Properties

The surface morphology of the membrane was observed by field emission scanning electron microscopy (FESEM, S4800, Hitachi, Tokyo, Japan). The composition of membrane contaminants was analyzed by Fourier transform attenuated total reflection infrared spectroscopy (ATR-FTIR, Thermo Scientific, Nicolet iS50, Madison, WI, USA). The water contact angle was measured by SL 200KB (Kino, Boston, MA, USA).

### 2.9. Data Processing

All experiments were performed in triplicate to ensure the reliability of the results, and the data were expressed as mean ± standard deviation. All statistical analyses were performed using Origin 2018 software.

## 3. Results and Discussion

### 3.1. Evaluation of Membrane Filtration Performance

#### 3.1.1. Evolution of Permeate Flux

**Figure 2:**
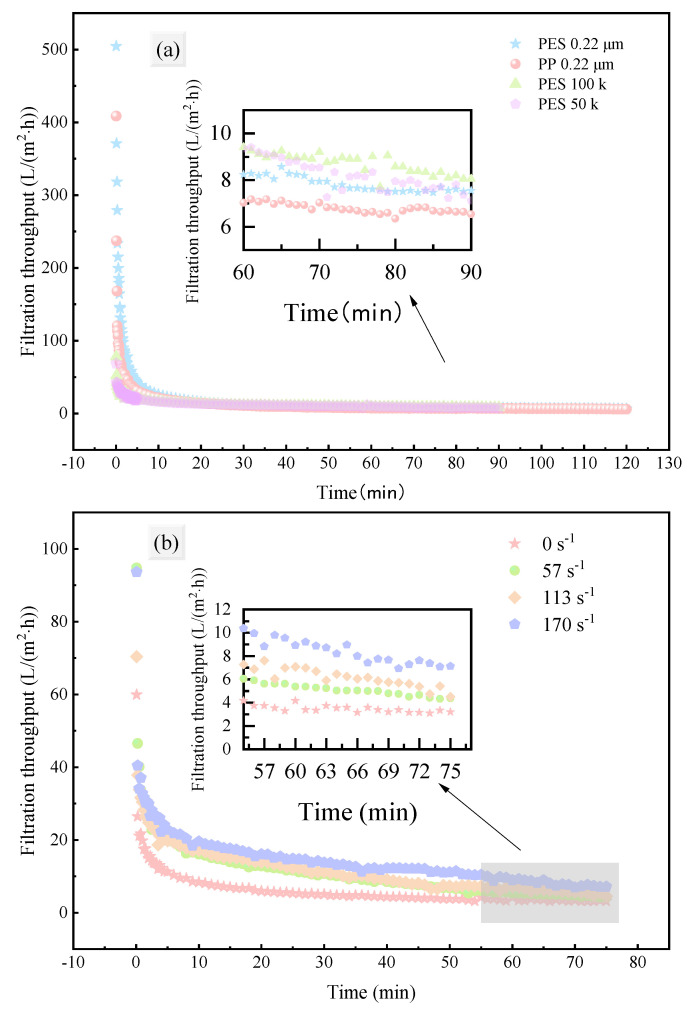
Variation in permeate flux during fermentation broth filtration under different operating conditions: (**a**) membrane material and pore size; (**b**) shear rate adjacent to the membrane.

This part investigated the effects of different membrane materials, pore sizes, and operating conditions (shear rate adjacent to the membrane) on the permeate flux during fermentation broth filtration. The variation in permeate flux over time was measured and plotted (Figure 2).

Despite differences in membrane materials, pore sizes, and operating conditions, the permeate flux exhibited a similar trend: a significant drop in the initial stage of filtration, followed by a gradual decrease and eventual stabilization. Such a sharp drop of permeate flux followed by stabilization is consistent with cake formation, which will be further discussed in Section 3.3. The initial flux of MF membranes was significantly higher than that of UF membranes, but the gap between them narrowed in the later stages of filtration and at steady-state flux. After 5 min of filtration, the flux decreased by 91.82%, 92.32%, 75.37%, and 68.64%, respectively; after 1 h of filtration, the flux only decreased by an additional 6.55%, 5.96%, 12.56%, and 17.73%, respectively. This result is consistent with previous studies [29], indicating that membrane material and pore size have a significant impact on initial flux, while their effect on steady-state flux is relatively small. Moreover, as shown in Figure 2a, the flux of PP membrane was the lowest in the later stage of filtration, while that of PES-100k membrane was the highest.

The effect of shear rate on permeate flux is shown in Figure 2b. Compared with static filtration (0 s^−1^), the shear force adjacent to the membrane (57, 113, 170 s^−1^) significantly enhanced the permeate flux, especially in the early and middle stages of filtration. However, as filtration progressed and membrane fouling (concentration polarization and pore blockage) intensified, the difference in flux between different shear rates gradually decreased. Nevertheless, the final steady-state flux was still increased by 84.04%, 101.38%, and 177.75% compared with static filtration, respectively.

#### 3.1.2. Filtration Efficiency Evaluation

**Figure 3:**
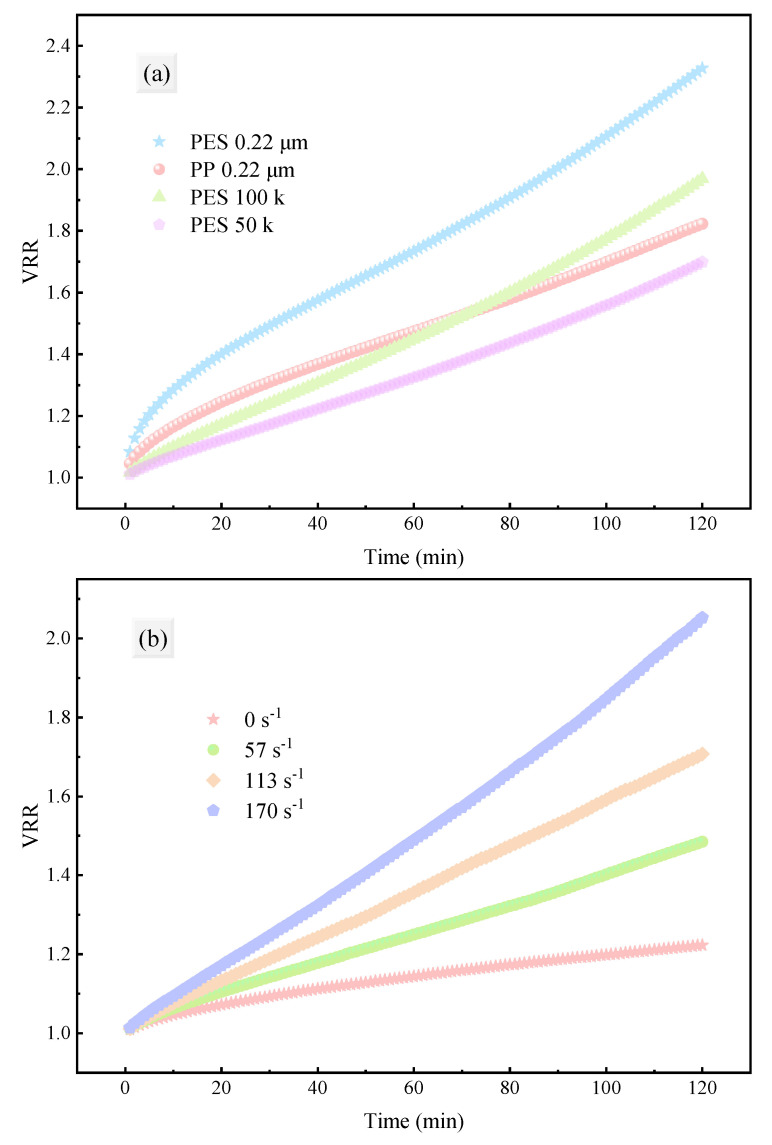
VRR over time during fermentation broth filtration under different operating conditions: (**a**) membrane material and pore size; (**b**) shear rate adjacent to the membrane.

Figure 3 shows the trend of VRR over time under different membrane types, pore sizes, and shear rates. As filtration progressed, the VRR increased steadily, indicating the continuous accumulation of filtrate volume. Among them, the PES-0.22 exhibited the highest filtration efficiency. Notably, PP-0.22 had a slightly higher filtration efficiency than the two UF membranes at the beginning of filtration due to its larger pore size. However, as filtration progressed, its efficiency was gradually surpassed by PES-100k membrane. This phenomenon may be closely related to the hydrophilicity/hydrophobicity of the materials. It should also be noted that the hydrophilicity of the PP membrane is much lower than that of the PES membrane (Table S1), indicating that the 1,3-PD fermentation broth is more suitable for treatment with a hydrophilic membrane, which is consistent with the results reported in previous studies [16,45]. Therefore, it is crucial to select a membrane material that matches the properties of the feed solution [46].

Furthermore, shear force adjacent to the membrane can effectively reduce concentration polarization in the dead-end filtration process [47]. As shown in Figure 3b, within the experimental range, the filtration efficiency increased approximately linearly with the increase in shear rate, implying that membrane fouling was mitigated. However, in practical industrial applications, the effect of continuously increasing shear rate on filtration efficiency will be limited, and excessive shear rate may lead to irreversible equipment damage, which is not conducive to long-term operation.

#### 3.1.3. 1,3-PD Recovery and Impurity Removal Rates

**Figure 4:**
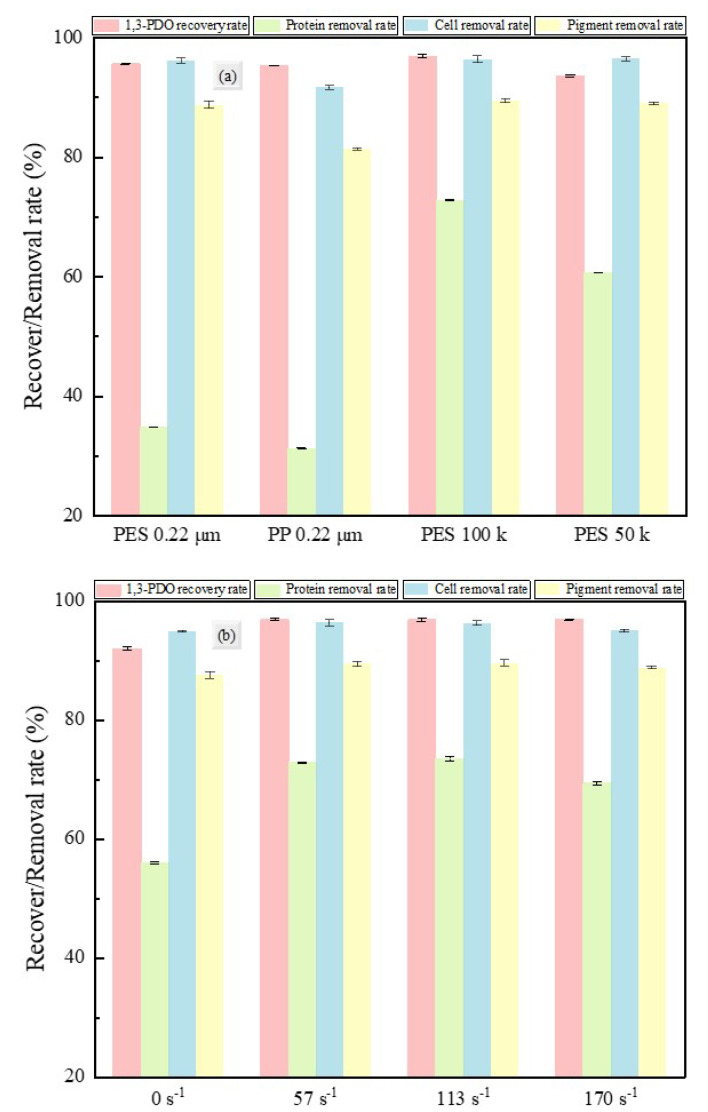
Removal rate of impurities after membrane separation under different conditions: (**a**) membrane material and pore size; (**b**) shear rate adjacent to the membrane.

This section focuses on the effects of different membrane materials, pore sizes, and shear rates on the filtration performance of fermentation broth, with specific evaluation indicators including the recovery rate of the target product (1,3-PD) in the permeate and the removal rates of major impurities (proteins, cells, and pigments). These data are the key basis for membrane selection. Figure 4 shows the recovery rates of 1,3-PD and the impurity removal rates after membrane separation under different conditions, and more results are detailed in Tables S2 and S3.

In terms of target product recovery, the PES-100k membrane achieved the highest recovery rate of 1,3-PD, at 96.95%, significantly reducing the retention loss of the target product. In terms of impurity removal, this membrane achieved a protein removal rate of 72.83%, which is significantly better than that of PES-0.22 and PP-0.22 membranes. Given that proteins can severely affect the subsequent separation and purification of 1,3-PD (e.g., forming an insoluble third phase in the extraction process, or forming azeotropes that increase separation difficulty and energy consumption, and causing product discoloration due to Maillard reactions at high temperatures [48]), it is crucial to efficiently remove proteins in the pretreatment stage. Moreover, the PES-100k membrane achieved excellent cell removal (96.30%) and pigment removal (89.38%), effectively reducing the burden on subsequent purification processes. In summary, the PES-100k membrane, while maintaining a high 1,3-PD recovery rate, effectively removed pigments and cell debris, improving product purity and emerging as the optimal choice for this separation process.

Additionally, the shear rates adjacent to the membrane had no significant effect on the recovery rate of 1,3-PD, cell removal rate, and pigment removal rate. Moderately increasing the shear rate can improve the protein removal rate, but excessively high shear rates can lead to a decrease in protein removal rate. This observation likely stems from the excessive shear rates thinning the filter cake to such an extent that small-molecular-weight proteins can more readily traverse the membrane medium. Therefore, continuously increasing the shear rate is not always beneficial for impurity removal.

### 3.2. Intrinsic Properties of Fermentation Broth

#### 3.2.1. Particle Properties

This section investigates the effects of different membrane materials, pore sizes, and shear rates on the particle size distribution, average particle size, and zeta potential of the fermentation broth before and after membrane filtration. These properties play a crucial role in the membrane filtration process [49,50].

**Figure 5:**
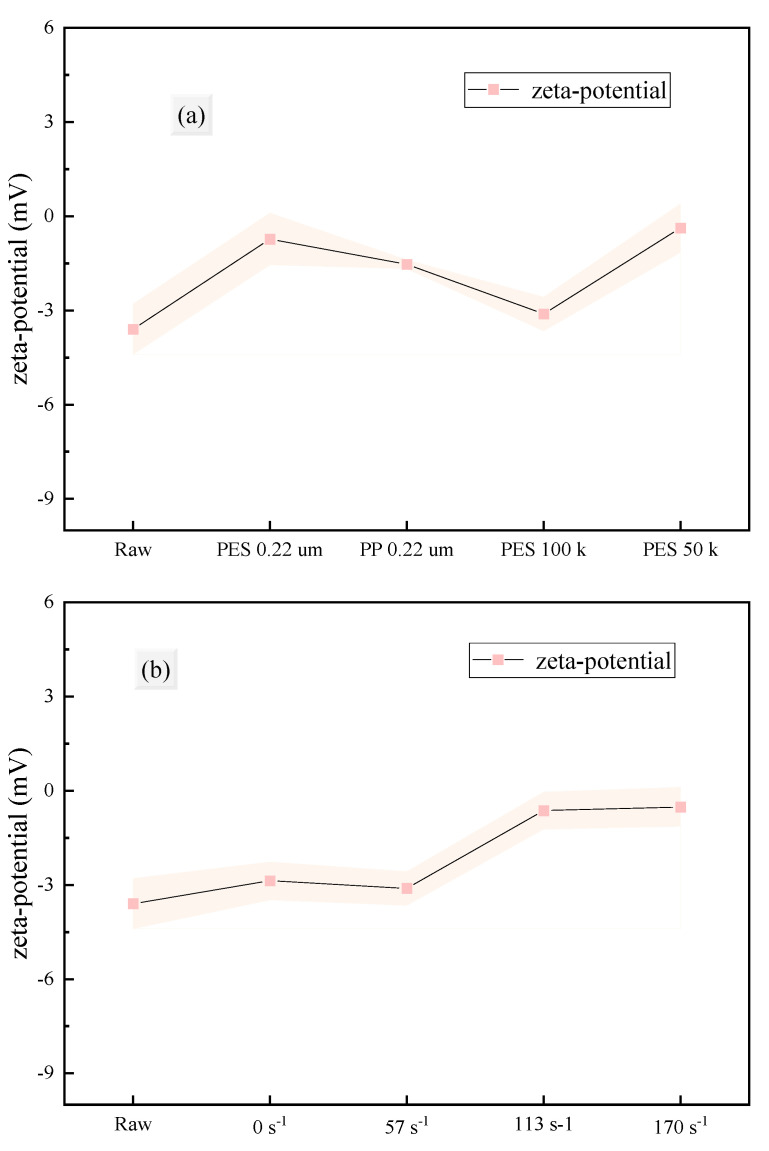
Zeta potential of fermentation broth after filtration under different conditions: (**a**) membrane material and pore size (**b**) shear rate adjacent to the membrane.

Zeta potential is an indicator of the repulsion or attraction between particles and is commonly used to evaluate the stability of colloidal systems [51,52]. A system with a high absolute zeta potential (>30 mV) is stable, while a system with a low absolute zeta potential (<5 mV) is unstable and prone to aggregation [53]. When the zeta potential is zero (isoelectric point), the system is the least stable. In this study, the absolute zeta potential of all fermentation broth samples was less than 5 mV (Figure 5), suggesting an unstable colloid where the colloidal particles were highly likely to aggregate rapidly into large molecular micelles. In comparison, the zeta potential of the filtrate after filtration with PES-100k membrane at a 57 s^−1^ shear rate had the highest absolute value, indicating the highest stability of the system. Additionally, increasing the shear rate also led to a decrease in the absolute zeta potential of the filtrate, reducing the colloidal stability of the system.

**Figure 6:**
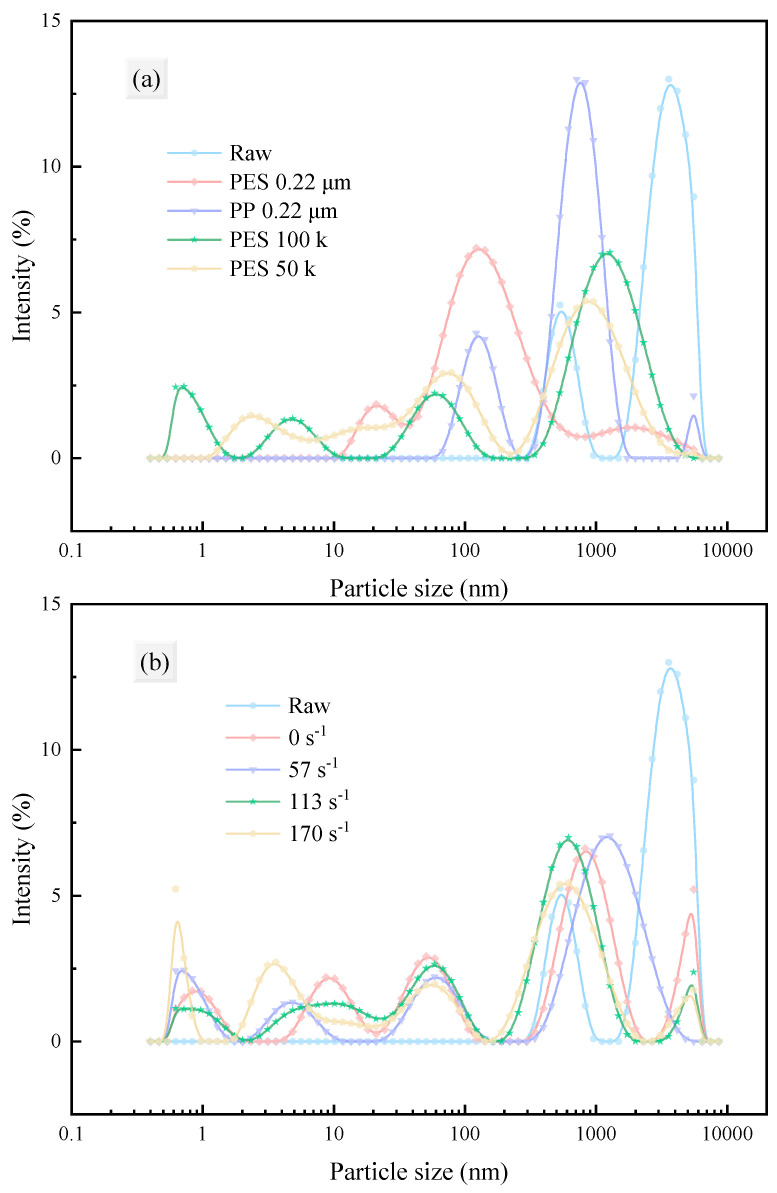
Particle size distribution of fermentation broth after filtration under different conditions: (**a**) membrane material and pore size; (**b**) shear rate adjacent to the membrane.

**Figure 7:**
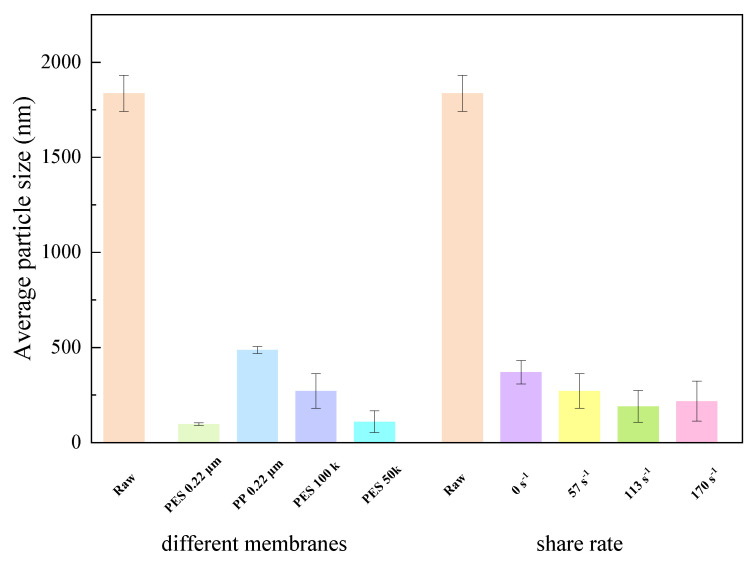
Average particle size of fermentation broth after filtration under different conditions.

**Figure 8:**
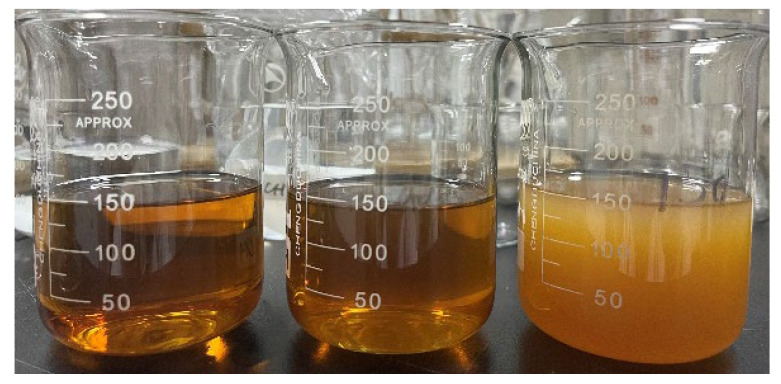
Comparison of the appearance of raw fermentation broth and filtrate: PES-0.22 (**left**), PP-0.22 (**center**), raw fermentation broth (**right**).

Furthermore, this section analyzes the average particle size and dispersity of the fermentation broth and filtrates under different conditions. Membrane filtration effectively removed large particles from the fermentation broth, significantly reducing the average particle size and increasing dispersity (polydispersity index, PdI). Figure 6 shows that there were differences in particle size distribution after filtration with different membranes, while the particle size distribution was similar for the same membrane at different shear rates. Notably, particles larger than the membrane pore size were still present in the filtrate, which is consistent with the zeta potential results (the system is unstable and prone to aggregation). The average particle size histogram (Figure 7) indicates that the average particle size was significantly reduced after filtration, removing most large particles, with little difference observed among different membrane materials, pore sizes, and shear rates. Interestingly, the average particle size after filtration with MF membranes was lower than that with UF membranes, possibly due to the low potential and instability of the system. Figure 8 visually demonstrates that the fermentation broth became clear and transparent after filtration.

#### 3.2.2. Rheological Properties

**Figure 9:**
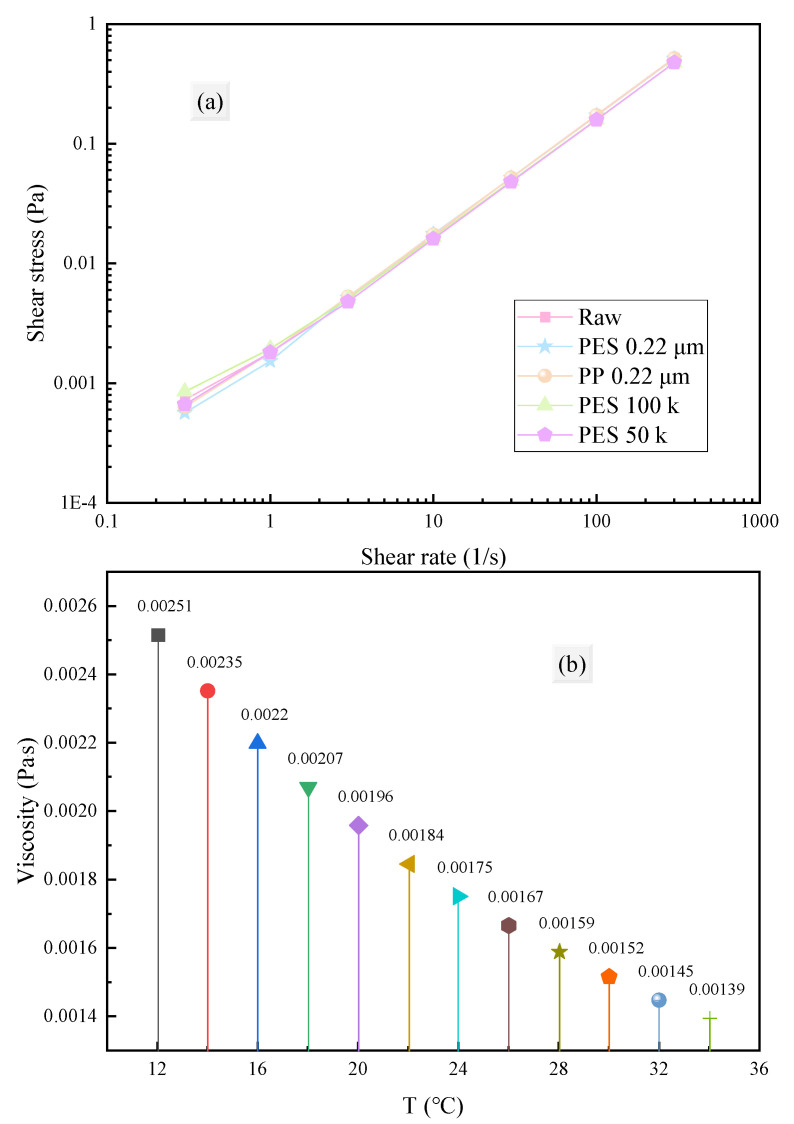
(**a**) Flow properties of the fermentation broth and the permeates under different conditions. (**b**) Viscosity variation in the fermentation broth over temperature.

As shown in Figure 9a, the fermentation broth exhibited shear-thinning behavior close to that of a Newtonian fluid. After treatment with membranes of different materials and pore sizes, there was no significant change in its flow characteristics, implying that the fermentation broth is minimally affected by external shear forces during processing.

Temperature has a significant impact on membrane filtration. Increasing temperature can reduce solution viscosity (Figure 9b) and increase permeate flux. This is mainly attributed to two factors: (1) reduced viscosity directly increases flux (Darcy’s law) [54]; (2) increased temperature raises the water vapor pressure at the membrane interface, enhancing the driving force for water transport [55]. Additionally, reduced viscosity improves fluidity, which can increase the filtration rate and reduce interactions within solute molecules as well as between solute molecules and the membrane surface, thereby alleviating membrane fouling [56]. However, in actual industrial applications, increasing the temperature of the filtration process will significantly increase energy consumption. Fortunately, the fermentation broth itself has a low viscosity (a 22.59% decrease in viscosity when the temperature increased from 20 °C to 30 °C), so there is no need to increase the temperature to reduce viscosity.

### 3.3. Membrane Fouling Mechanism

#### 3.3.1. Resistance Distribution

**Figure 10:**
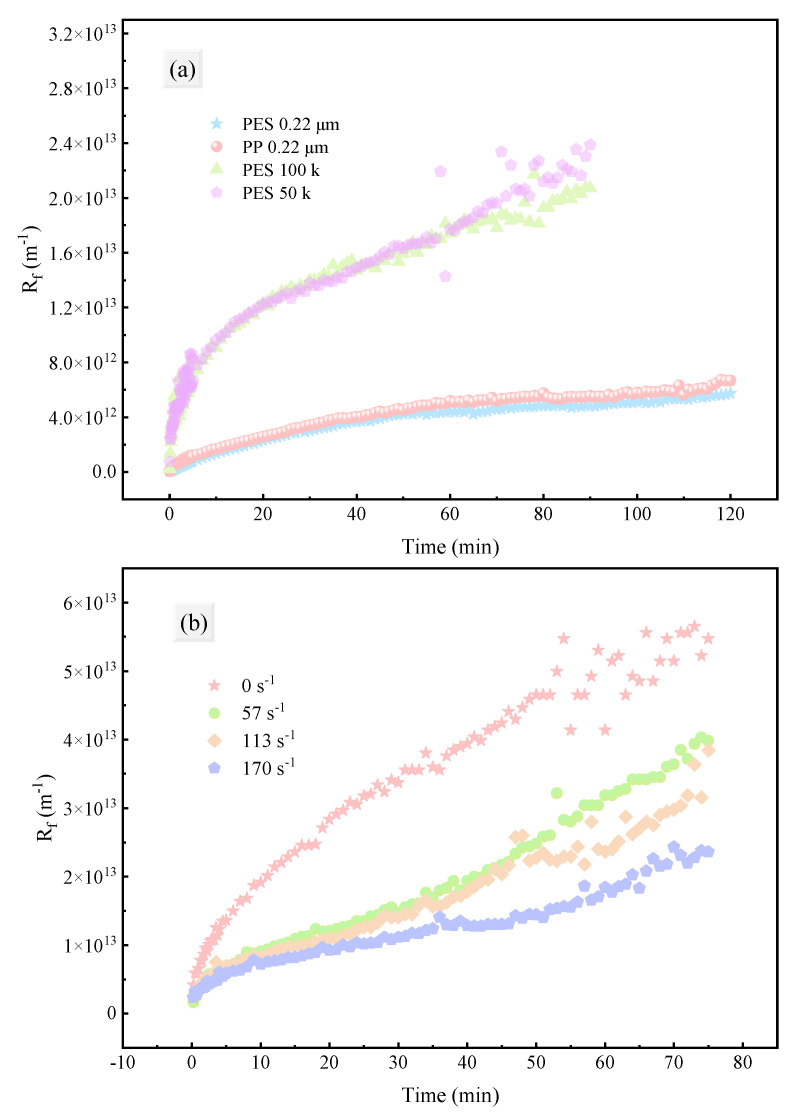
Increasing trend of fouling resistance ($R_{f}$) during fermentation broth filtration under different conditions: (**a**) membrane material and pore size; (**b**) shear rate adjacent to the membrane.

The membrane fouling resistance ($R_{f}$) continuously increased with filtration time (Figure 10). The trends for UF and MF membranes were similar: a rapid increase in the early stage followed by a slower growth. However, the fouling resistance and its growth rate of UF membranes were significantly higher than those of MF membranes. This might be related to flux and pore size: in the early stage of filtration, high flux and strong convection led to more macromolecular substances being rapidly brought to the membrane surface and retained, resulting in rapid thickening of the fouling layer or concentration polarization layer and thus a rapid increase in the total process resistance ($R_{t}$). Additionally, the smaller pore size of UF membranes made them more prone to pore blockage, leading to significantly higher fouling resistance compared to MF membranes.

**Figure 11:**
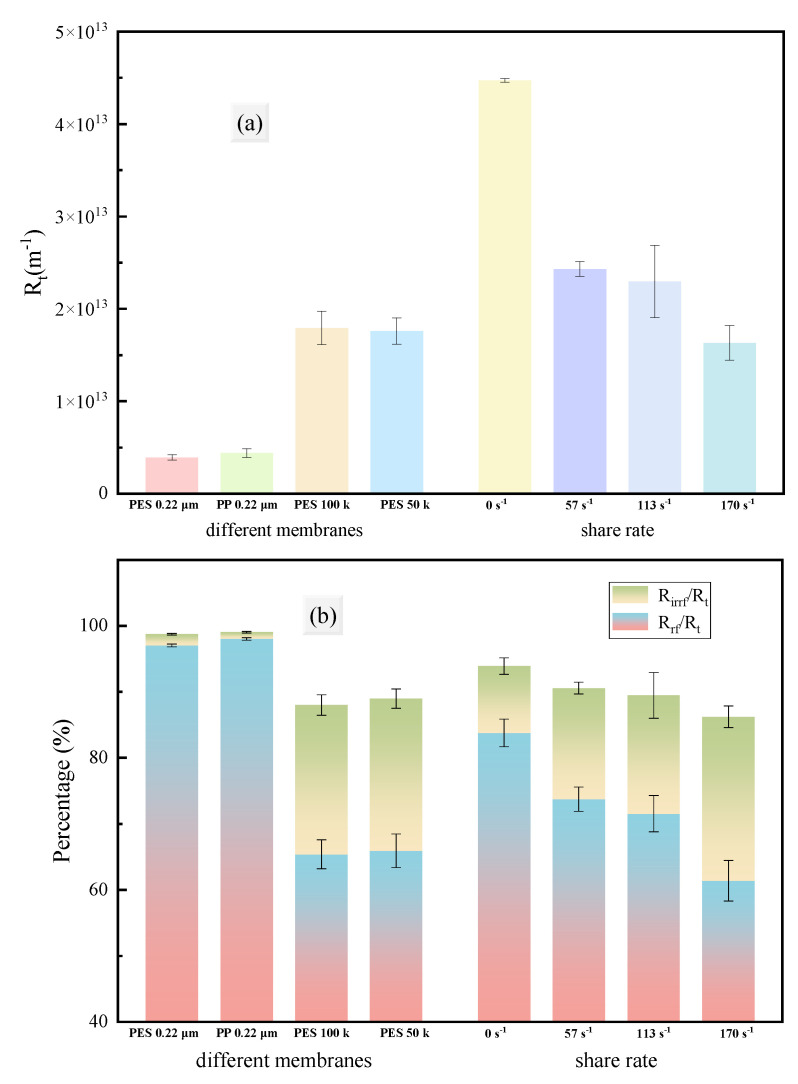
Total resistance (**a**) and resistance distribution (**b**) of fermentation broth under different conditions of filtration.

Increasing the shear rate at the membrane surface effectively inhibited the growth of total process resistance ($R_{t}$), but the inhibitory effect of excessively high shear rates was limited (Figure 10b, Figure 11). This might be related to the properties of the fouling layer: increasing the shear force on the membrane surface effectively suppressed concentration polarization and controlled fouling layer accumulation; however, the turbulence caused by stirring mainly destroyed dense or weakly adherent fouling layers, with little effect on strongly adherent fouling layers, so further increasing the shear rate could not continuously reduce the total resistance.

Analysis of the resistance composition (Figure 11b) indicated that the reversible resistance ($R_{rf}$) of MF membranes accounted for a high proportion, while the irreversible resistance ($R_{irrf}$) of UF membranes accounted for a high proportion. Increasing the shear rate decreased the proportion of reversible resistance, indicating that the shear effect reduced the total resistance mainly by disrupting the reversible fouling layer. The increase in the proportion of irreversible resistance was due to its value remaining essentially unchanged while the total resistance decreased. In summary, increasing the shear rate did not significantly affect the irreversible resistance of the membrane separation process, but mainly increased the permeate flux by reducing the reversible resistance.

#### 3.3.2. Fouling Propensity

**Figure 12:**
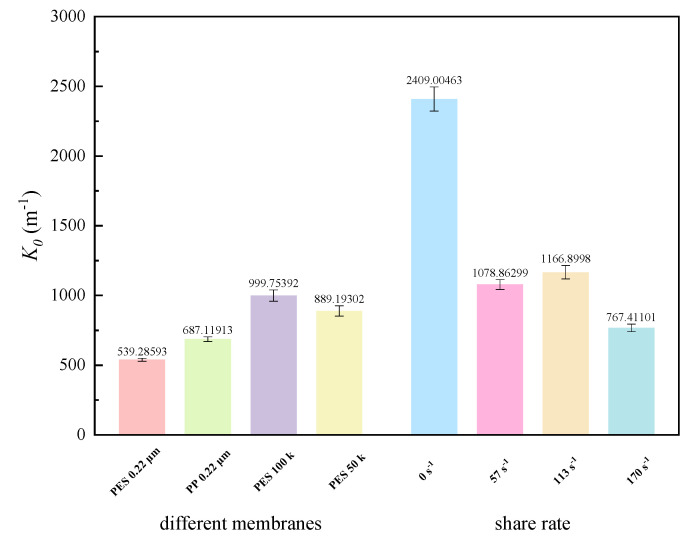
Fouling propensity ($K_{0}$) of fermentation broth filtration under different conditions.

By fitting the experimental data, reliable values of the fouling propensity coefficient ($K_{0}$) were obtained (Figure 12). $K_{0}$ characterizes the rate of fouling resistance growth (i.e., the growth rate of the fouling layer), and its value is closely related to membrane materials, pore sizes, and operating conditions (shear rate adjacent to the membrane). Although PES-0.22 and PP-0.22 had the same pore size, the $K_{0}$ of the PP membrane was significantly higher, indicating that the PP membrane was more prone to fouling during filtration of this fermentation broth. Due to the significantly smaller pore size of UF membranes compared to MF membranes, their $K_{0}$ values were also significantly higher. For UF membranes, increasing the shear rate significantly reduced $K_{0}$ ($K_{0}$ was extremely high in the absence of shear), and the $K_{0}$ values at 57 s^−1^ and 113 s^−1^ were similar, with only a slight decrease at 170 s^−1^. Such results suggest that increasing shear rates adjacent to the membrane effectively suppressed the growth rate of the fouling layer.

#### 3.3.3. Membrane Pore Blocking Mechanism

**Figure 13:**
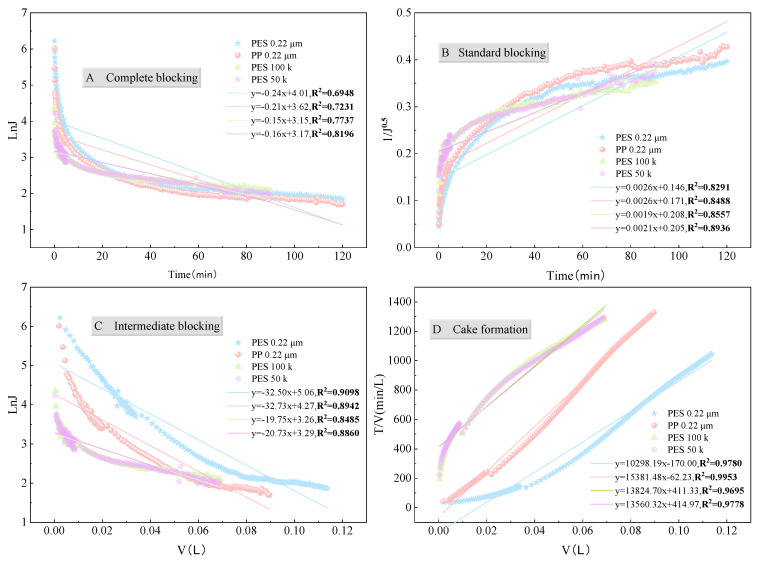
Fitting curves of Hermia model to experimental data of fermentation broth filtration under different membrane materials and pore sizes: (**A**) Complete blocking (**B**) Standard blocking (**C**) Intermediate blocking (**D**) Cake formation.

**Figure 14:**
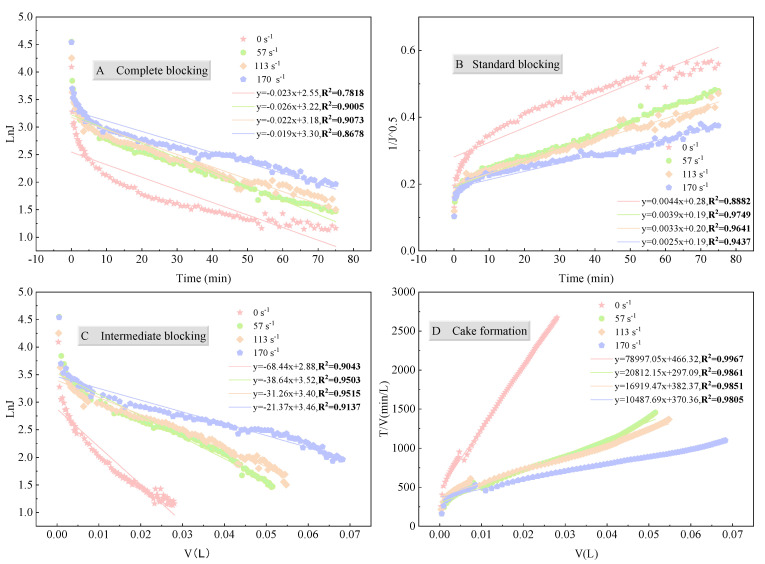
Fitting curves of Hermia model to experimental data of fermentation broth filtration under different shear rates: (**A**) Complete blocking (**B**) Standard blocking (**C**) Intermediate blocking (**D**) Cake formation.

The fitting results (Figure 13 and Figure 14, Table S4) show that, regardless of the membrane type, pore size, or shear rate, the experimental data best fit the cake filtration model, although other models also exhibited some degree of fit under certain conditions. The Hermia model indicates that the blocking mechanism is closely related to the particle size distribution of the feed solution. In this study, most particles in the fermentation broth were larger than the membrane pore size. As filtration progressed, the pollutants accumulated on the membrane surface to form a compressible cake layer, which was the main cause of increased membrane fouling [57,58,59]. It should also be noted that the Hermia model constants of the two PES membranes are comparable, while that of the PP membrane is the highest (Figure 13D), implying a faster cake growth and a more pronounced decline in permeate flux for PP membrane, which is consistent with the results presented in Section 3.1.

Given the relatively low fit of the cake filtration model in the initial stage of filtration (Table S4), it is inferred that the blocking mechanism is not singular but rather a combination of multiple mechanisms. Therefore, the data were fitted in segments (first 2 min, 2–5 min, 0–5 min, and the entire process). Table S4 shows that, even in the initial stage (0–2 min), the PP membrane primarily followed the cake filtration model. In contrast, the PES membrane exhibited a certain degree of correlation with all four mechanisms in the initial stage, indicating that the rapid decline in initial flux was a result of the combined action of multiple mechanisms. Nevertheless, the overall blocking mechanism of the PES membrane was still dominated by cake filtration [60].

In summary, the fouling behavior of the PES membrane during filtration of the fermentation broth can be inferred as follows: initially, small hydrophilic molecules entered the membrane pores, causing standard blocking, while large molecules formed complete blocking on the membrane surface; subsequently, standard blocking rapidly transformed into intermediate blocking; as particles accumulated on the membrane surface, a compressible cake layer quickly formed, transitioning the process into the cake filtration stage. In contrast, the PP membrane primarily followed the cake filtration mechanism throughout the filtration process. The main reason for this difference is the membrane material: as mentioned before, the PP membrane was less hydrophilic than the PES membrane (Table S1). Therefore, the hydrophilic particles in the aqueous fermentation broth are more likely to form a cake layer on the membrane surface rather than enter the membrane pores during the initial stage of filtration. This is why the proportion of irreversible membrane fouling for the PP membrane was indeed smaller than that for the PES membrane (Figure 11b).

### 3.4. Foulants Identification

**Figure 15:**
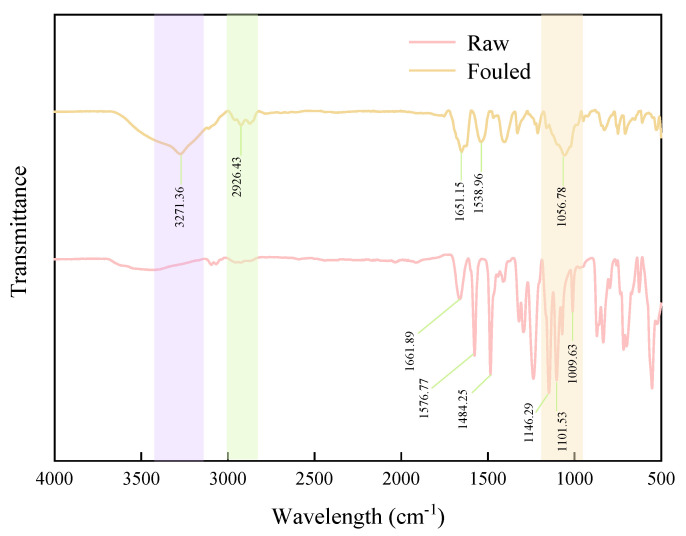
ATR-FTIR spectra of PES-100k membrane on different membrane conditions.

Morphology of the fouled PES-100k membrane was revealed by SEM, showing an obvious cake deposited layer on the membrane surface (Figure S1). The surface of the PES-100k original membrane and the fouled membrane was then characterized using ATR-FTIR technology, and the infrared spectroscopy results are shown in Figure 15. A comparison of the two spectra reveals that the fouled membrane exhibits distinct characteristic absorption peaks at 3271.36 cm^−1^, 2936.43 cm^−1^, 1651.15 cm^−1^, and 1538.96 cm^−1^, which correspond to N–H stretching vibration, C–H stretching vibration, amide I band, and amide II band, respectively. Collectively, these characteristics indicate that the contaminants on the membrane surface contain proteinaceous substances. The original membrane shows characteristic peaks caused by aromatic C=C skeleton vibration at wavenumbers such as 1661.89 cm^−1^, 1576.77 cm^−1^, and 1484.25 cm^−1^, which is consistent with the characteristic absorption of PES near 1577 cm^−1^ and 1484 cm^−1^ reported in the literature [61]. In addition, an absorption peak possibly attributed to the C–O–C stretching vibration of polysaccharides is observed at 1056.78 cm^−1^ in the fouled membrane. However, due to the presence of ether bond structures in PES itself, there is also a C–O stretching vibration peak at a similar wavenumber. Therefore, it cannot be clearly determined whether the absorption peak at this position originates from polysaccharides in the contaminants. Except for the above differences, the remaining peak positions in the two spectra are basically consistent and can be attributed to the PES membrane material itself. Notably, the intensity of the characteristic peaks of the fouled membrane is generally weaker, which may be due to the coverage of the membrane surface by a contaminant layer from the fermentation broth, resulting in a reduction in the penetration depth of the infrared beam and a decrease in the attenuated total reflection signal intensity.

## 4. Conclusions

This work systematically investigated the membrane separation process of 1,3-PD fermentation broth, focusing on membrane filtration performance, intrinsic properties of the fermentation broth, and membrane fouling mechanisms, providing a significant reference for the selection of membrane modules in industrial applications of clarification of 1,3-PD fermentation broth.

Results show that the 1,3-PD fermentation broth was a shear-thinning fluid close to Newtonian behavior, with minimal changes in flow properties after membrane treatment. Membrane filtration effectively removed large particles, reducing the average particle size and increasing the system stability of the broth. The filtration of fermentation broth was mainly dominated by cake formation, and the main foulant was identified as proteinaceous substances. The PES-100k membrane exhibited the best overall performance: under appropriate shear action on the membrane surface, it had a relatively low membrane fouling propensity, with a high recovery rate of 1,3-PD and excellent removal effects on impurities, showing an optimal trade-off between recovery and fouling control.

In conclusion, this study provided comprehensive theoretical support for optimizing the membrane separation process of 1,3-PD fermentation broth, offering an important theoretical basis and data support for industrial membrane separation process material selection, parameter optimization, and fouling control.

## Figures and Tables

**Table 1:** The application of membrane separation technology in 1,3-PD fermentation broth.

Reference	Membrane Separation Technology	Research Content (Membrane-Related)	Research on Membrane Fouling Mechanism
[15]	NF and MD	Separation efficiency and process optimization	Mentioned but not thoroughly investigated
[9,12,13,14]	UF	Membrane filtration as a pre-treatment step in downstream processing	None
[11]	MF		
[16]	MF	Macroscopic performance (flux, turbidity) and cleaning methods	Qualitative description via SEM
[20]	UF	Optimization of operational parameters (TMP, feed flow rate Q, feed pH)	Resistance-in-series analysis
[22]	UF	Integration of bioreactor with ceramic membrane to product 1,3-PD	None
[24]	RO	Exploration on rejection characteristics and antifouling performance of RO for 1,3-PD fermentation broth	None
[25]	NF and RO	Investigation of applicability of NF and RO to separate 1,3-PD fermentation broth	None
[26]	MF	Combination of ceramic membrane filtration with membrane extraction to improve production of 1,3-PD	None

## Supplementary Materials

The following supporting information can be downloaded at: https://www.mdpi.com/article/10.3390/membranes15090276/s1, Figure S1: SEM images of PES 100 k ultrafiltration membranes (a) Raw (b) Washed (c) Fouled; Table S1: Detailed Information of Membrane Materials; Table S2: Content of components in fermentation broth and removal rate of impurities after treatment under different membrane material and pore size; Table S3: Content of components in fermentation broth and removal rate of impurities after treatment under different shear rate adjacent to the membrane; Table S4: R^2^ values of Hermia pore blocking models with piecewise fitting under different conditions.
