# Supplementary material for: Fouling Mechanisms in the Clarification of 1,3-Propanediol Fermentation Broths by Membrane Processes
Source: Membranes (Basel). 2025 Sep 12;15(9):276. doi: 10.3390/membranes15090276 (PMC12471324; doi:10.3390/membranes15090276)
Supplement: Supplementary file 1 [file membranes-15-00276-s001.zip › membranes-3843110-supplementary.pdf]

Supplementary data

Table S1: Detailed Information of Membrane Materials

Denote	Batch No./Model	Material	Pore Size	Hydrophilicity/ Hydrophobicity	Thickness(μm)	MWCO(kD)	Contact angle(°)	Water flux(L/(m²·h)) (25°C)	Supplier
PES-0.22	KZ20230527102	Polyethersulfone	0.22 μm	Hydrophilic	120±10		45.6±5.6	1487.93±60.89[a]	Haining Kezhun Co., Ltd.
PP-0.22	KZ20230527101	Polypropylene	0.22 μm	Hydrophilic	135±10		83.7±3.7	1692.78±129.31[a]	Haining Kezhun Co., Ltd.
PES-100k	MSC80100	Polyethersulfone	100 k	Hydrophilic	150±10	100	42.4±8.2	187.51±15.34[b]	Shanghai Mosu Co., Ltd.
PES-50k	MSC80050	Polyethersulfone	50 k	Hydrophilic	150±10	50	40.8±6.5	168.74±12.71[b]	Shanghai Mosu Co., Ltd.

Note: Some data is provided by the corresponding manufacturer. "a" represents the pure water flux measured at 0.02 MPa, "b" represents the pure water flux measured at 0.1 MPa.

Table S2: Content of components in fermentation broth and removal rate of impurities after treatment under different membrane material and pore size

	1,3-PDO (g/L)	Protein (g/L)	Average particle size (nm)	1,3-PDO Recovery rate	Protein removal rate	Cell removal rate	Pigment removal rate
Raw	82.80±0.65	0.4311±0.0039	1836.667±94.13				
PES-0.22	79.72±0.85	0.2671±0.0056	97.715±7.14	0.9565±0.011	0.3483±0.0033	0.9604±0.0014	0.8867±0.0017
PP-0.22	80.23±0.99	0.2559±0.0099	486.967±18.72	0.9538±0.012	0.3128±0.0027	0.9156±0.0024	0.8131±0.011
PES-100k	79.85±0.82	0.1616±0.0036	271.033±91.34	0.9695±0.015	0.7283±0.0073	0.9630±0.0079	0.8938±0.014
PES-50k	77.26±0.68	0.1241±0015	109.546±57.14	0.9361±0.0054	0.6069±0.0040	0.9635±0.0029	0.8893±0.0053

Note: Share rate is 57 s^{-1}.

Table S3: Content of components in fermentation broth and removal rate of impurities after treatment under different shear rate adjacent to the membrane

	1,3-PDO (g/L)	Protein (g/L)	Average particle size (nm)	1,3-PDO Recovery rate	Protein removal rate	Cell removal rate	Pigment removal rate
Raw	82.80±0.65	0.4311±0.0039	1836.667±94.13				
PES 0 s^{-1}	74.75±0.83	0.2101±0.0015	369.933±61.85	0.9206±0.016	0.5598±0.0034	0.9484±0.011	0.8751±0.013
PES 57 s^{-1}	79.85±0.82	0.1616±0.0032	271.033±91.34	0.9695±0.015	0.7283±0.0073	0.9630±0.0079	0.8938±0.014
PES 113 s^{-1}	78.36±0.77	0.1647±0.0036	190.118±83.57	0.9693±0.014	0.7350±0.0083	0.9623±0.0084	0.8953±0.013
PES 170 s^{-1}	79.53±1.01	0.1696±0.0028	217.567±105.43	0.9693±0.0048	0.6943±0.0064	0.9497±0.018	0.8877±0.015

Note: All membranes are PES 100 k ultrafiltration membranes

Table S4: R^2 values of Hermia pore blocking models with piecewise fitting under different conditions

	Time period	0 s^{-1}	57 s^{-1}	113 s^{-1}	170 s^{-1}	PES 0.22 µm	PP 0.22 µm	PES 100 k	PES 50 k
Complete blocking	0-2 min	0.6614	0.7067	0.6850	0.5089	0.9307	0.7866	0.5982	0.6903
	2-5 min	0.9071	0.8358	0.6935	0.7849	0.8982	0.8740	0.4877	0.4832
	0-5 min	0.6841	0.6561	0.6885	0.7610	0.8772	0.7600	0.6714	0.7365
	all	0.7818	0.9005	0.9073	0.8678	0.6948	0.7231	0.7737	0.8196
Standard blocking	0-2 min	0.7504	0.7882	0.7549	0.5674	0.9788	0.8952	0.6276	0.7309
	2-5 min	0.9096	0.8410	0.6799	0.7814	0.9093	0.8828	0.4906	0.4762
	0-5 min	0.8007	0.7571	0.7704	0.6792	0.9515	0.8833	0.7278	0.7715
	all	0.8882	0.9749	0.9641	0.9437	0.8291	0.8488	0.8557	0.8936
Intermediate blocking	0-5 min	0.7640	0.7071	0.6625	0.6271	0.9846	0.8917	0.7133	0.7680
	all	0.9043	0.9503	0.9515	0.9137	0.9098	0.8942	0.8485	0.8860
Cake formation	0-5 min	0.9987	0.9854	0.9888	0.9947	0.9888	0.9962	0.9713	0.9801
	all	0.9767	0.9861	0.9851	0.9805	0.9780	0.9953	0.9695	0.9778

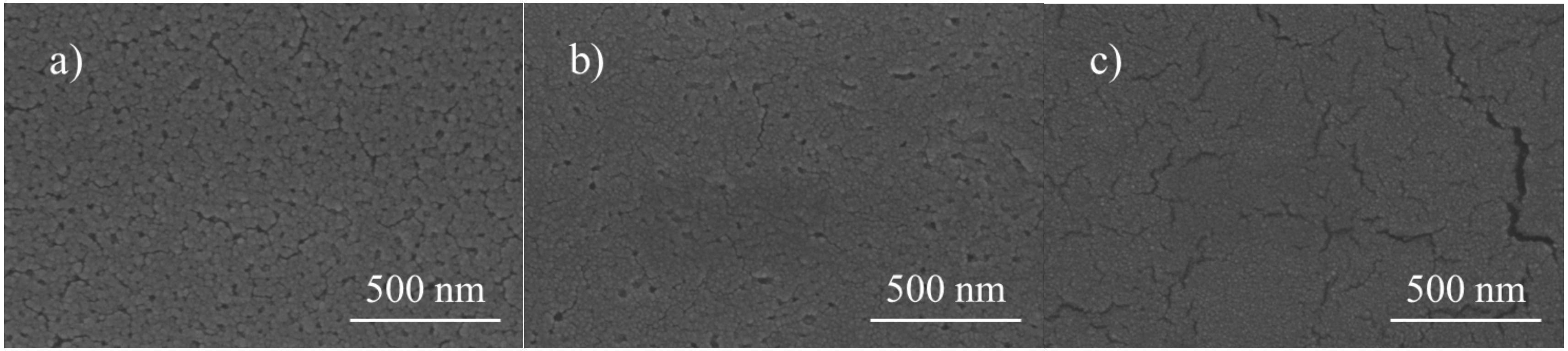

Figure S1. SEM images of PES 100 k ultrafiltration membranes (a) Raw (b) Washed (c) Fouled
